# Assessing Ploidy Level Analysis and Single Pollen Genotyping of Diploid and Euploid Citrus Genotypes by Fluorescence-Activated Cell Sorting and Whole-Genome Amplification

**DOI:** 10.3389/fpls.2019.01174

**Published:** 2019-09-24

**Authors:** Miguel Garavello, José Cuenca, Steven Dreissig, Jörg Fuchs, Andreas Houben, Pablo Aleza

**Affiliations:** ^1^Centro de Citricultura y Producción Vegetal, Instituto Valenciano de Investigaciones Agrarias (IVIA), Moncada, Valencia, Spain; ^2^INTA, Concordia Agricultural Experiment Station, Concordia, Argentina; ^3^Department of Breeding Research, Leibniz Institute of Plant Genetics and Crop Plant Research (IPK) Gatersleben, Seeland, Germany

**Keywords:** flow cytometry, triploid, tetraploid, fluorescence-activated cell sorting, whole genome amplification, SSR and SNP markers, unreduced gametes

## Abstract

Flow cytometry is widely used to determine genome size and ploidy level in plants. This technique, when coupled with fluorescence-activated cell sorting (FACS), whole genome amplification and genotyping (WGA), opens up new opportunities for genetic studies of individualized nuclei. This strategy was used to analyze the genetic composition of single pollen nuclei of different citrus species. The flow cytometry and microscope observations allowed us to differentiate the populations of pollen nuclei present in the diploid and euploid genotypes analyzed, showing that citrus has binuclear pollen. We have identified in the “CSO” tangor an additional nuclei population composed by the vegetative plus generative nuclei. Genotyping of this nuclei population revealed that vegetative and generative nuclei show the same genetic configuration. In addition, we have demonstrated the presence of unreduced gametes in the diploid genotype “Mexican lime.” Genomic amplification is a robust method for haploid nuclei genotyping with several molecular markers, whereas in diploid nuclei using heterozygous markers showed a bias towards one of the two alleles, limiting the use of this tool in this type of nuclei. We further discuss the importance and applications of single pollen genotyping in citrus genetic studies.

## Introduction

Flow cytometry has become a widely used technique for genome size estimation and ploidy analysis in plant research because of its high throughput, accuracy and resolution as well as low operating cost per sample. Fluorescence-activated cell sorting (FACS) enables the separation of nuclei according to their optical properties. The direct analysis of individual nuclei is generally limited by their low amount of DNA (Hou et al., 2015). To circumvent this problem Whole Genome Amplification (WGA) can be applied. WGA is a method for the robust amplification of a complete genome, starting with nanogram DNA quantities and resulting in microgram quantities of the amplified products (de Bourcy et al., 2014; Dreissig et al., 2015; Gawad et al., 2016). A critical step in WGA is the minimization of the amplification bias, generation of mutations and chimeras. In this sense, isothermal methods such as Multiple Displacement Amplification (MDA) have demonstrated to introduce a low error rate (Gawad et al., 2016).

Beside the application of leaves, young stems, flowers, roots, and seeds, also mature pollen grains collected from anthers of several herbaceous and woody species have been used for flow cytometry analysis (Van Tuyl et al., 1989; Bino et al., 1990; Zhang et al., 1992; Mishiba et al., 2000; Pichot and El Maâtaoui, 2000; Pan et al., 2004; Stehlik et al., 2007; Kron and Husband, 2012; Chung et al., 2013; Dreissig et al., 2017). Angiosperm pollen contains both vegetative and generative sperm nuclei, which can be structurally and morphologically different (Van Tuyl et al., 1989; Bino et al., 1990; Dewitte et al., 2009). The genotyping of individualized pollen grain nuclei opens up new opportunities in different areas of research such as the ecology of pollination, genetic, and genomic studies (Isagi and Suyama, 2010). In addition, genotyping of individual pollen grains can be useful for the determination of the haplotypes of the male parent and meiotic recombination patterns (Mase et al., 2014; Dreissig et al., 2017) and also allows performing studies on the genetic structures of pollen grain populations as compared with those originated at the plant level, without interferences due to a potential cross-incompatibility (Gu et al., 2013). However, to our knowledge, ploidy level analysis and genotyping of pollen grains have not been previously assessed in citrus.

Diploid genotypes are the most common one in *Citrus* and related genera, with a basic chromosome number of x = 9 (Krug, 1943); although euploids and aneuploids have been induced or found occasionally, with triploids and tetraploids being the most common euploid variations (Lee, 1988). The genus *Citrus* can be used as a model for the study of somatic and sexual polyploidization (Ollitrault et al., 2008; Aleza et al., 2009a; Aleza et al., 2009b; Cuenca et al., 2015a; Aleza et al., 2016). Sexual polyploidization by the formation of female unreduced gametes is a relatively frequent event in citrus and it is routinely used to obtain triploid hybrids through hybridizations between diploid progenitors (Aleza et al., 2010; Cuenca et al., 2011; Cuenca et al., 2015a; Cuenca et al., 2015b; Rouiss et al., 2017b). The formation of unreduced pollen grains in citrus has been also reported from the genetic analysis of tetraploid populations (Rouiss et al., 2017a) and the direct observation and hand-made isolation of large pollen grains of one diploid genotype, associated with unreduced gametes (Honsho et al., 2016). In order to improve the efficiency of citrus triploid breeding based on sexual hybridizations between diploid parents, it would be of great interest to develop a simple methodology that allows the ploidy level analysis of mature pollen grains for identifying parents producing unreduced pollen grains. In addition, this methodology would also increase the knowledge about the viability of citrus triploid hybrids pollen grains.

On the other hand, there is a lack of knowledge about citrus pollen genotyping. A limiting factor of this technique is the small amount of DNA per pollen and, thus, limiting the number of markers that can be used for analyzing single pollen grains (Matsuki et al., 2007; Martin et al., 2019). Citrus pollen genotyping has been previously only performed by multiplex PCR (Honsho et al., 2016). In the present study, we describe an effective methodology to determine the ploidy level in mature pollen grains of diploid, triploid, and tetraploid citrus genotypes, using FACS, followed by WGA and genotyping of individualized nuclei from pollen grains by Simple Sequence Repeats (SSR) and Single Nucleotide Polymorphism (SNP) molecular markers. Finally, we discuss the applications and implications of flow cytometry to determine the ploidy level of citrus pollen grains populations and the use of FACS combined with WGA for the genotyping of individualized nuclei.

## Materials and Methods

### Plant Material

A total of 59 genotypes of different *Citrus* species and ploidy levels (1 haploid, 45 diploid, 7 triploid, and 6 tetraploid genotypes) were used from the pathogen-free Germplasm Bank (Navarro et al., 2002) (**Table 1**) of the Instituto Valenciano de Investigaciones Agrarias (IVIA) located at Moncada (Valencia, Spain).

**Table 1:** Citrus genotypes used to measure the relative DNA content of pollen grains by flow cytometry.

Ploidy level	Genotype	*Species	Bank ID
1X	Haploid clementine	*C. clementine*	IVIA-638
2X	N´15 mandarin	*C. hybrid*	–
2X	CSO tangor	*C. hybrid*	–
2X	Hamlin sweet orange	*C. sinensis*	IVIA-010
2X	Pineapple sweet orange	*C. sinensis*	IVIA-011
2X	Wilking mandarin	*C. reticulate*	IVIA-028
2X	Sanguinelli blood orange	*C. sinensis*	IVIA-034
2X	Fina clementine	*C. clementine*	IVIA-039
2X	Fino 74-L-08 lemon	*C. limon*	IVIA-049
2X	Verna lemon	*C. limon*	IVIA-062
2X	Fortune mandarin	*C. reticulate*	IVIA-080
2X	Temple tangor	*C. temple*	IVIA-081
2X	Fairchild mandarin	*C. reticulate*	IVIA-083
2X	Seville sour orange	*C. aurantium*	IVIA-117
2X	Olinda sweet orange	*C. sinensis*	IVIA-127
2X	Mexican lime	*C. aurantifolia*	IVIA-164
2X	Marsh grapefruit	*C. paradise*	IVIA-176
2X	Campeona mandarin	*C. nobilis*	IVIA-193
2X	Ellendale tangor	*C. reticulate*	IVIA-194
2X	Murcott tangor	*C. reticulate*	IVIA-196
2X	Star Ruby grapefruit	*C. paradise*	IVIA-197
2X	Fingered citron	*C. medica*	IVIA-202
2X	Chandler pummelo	*C. maxima*	IVIA-207
2X	Limoneira Lisbon lemon	*C. limon*	IVIA-214
2X	Frost navel orange	*C. sinensis*	IVIA-222
2X	Tachibana	*C. tachibana*	IVIA-237
2X	Duncan grapefruit	*C. paradise*	IVIA-274
2X	Pink pummelo	*C. maxima*	IVIA-275
2X	Ortanique tangor	*C. reticulate*	IVIA-276
2X	Río Red grapefruit	*C. paradise*	IVIA-289
2X	Eureka Frost lemon	*C. limon*	IVIA-297
2X	Palestine sweet lime	*C. limettioides*	IVIA-305
2X	Gil pummelo	*C. maxima*	IVIA-321
2X	Bernalina sweet orange	*C. sinensis*	IVIA-331
2X	Rough lemon	*C. jambhiri*	IVIA-333
2X	Rangpur lime	*C. limonia*	IVIA-334
2X	Seminole tangelo	*C. hybrid*	IVIA-348
2X	Willow leaf mandarin	*C. deliciosa*	IVIA-383
2x	Carrizo citrange	*C. sinensis x P. trifoliata*	IVIA-387
2X	Anana mandarin	*C. reticulata*	IVIA-390
2X	Tarocco Rosso blood orange	*C. sinensis*	IVIA-392
2X	Moncada mandarin	*C. hybrid*	IVIA-421
2X	Alemow	*C. macrophylla*	IVIA-518
2X	Corsican citron	*C. medica*	IVIA-567
2X	Imperial mandarin	*C. reticulata*	IVIA-576
2X	Nadorcott mandarin	*C. reticulata*	IVIA-641
3X	CidMexT 99-7	*C. hybrid*	–
3X	Oroblanco	*C. maxima x C. paradisi*	IVIA-302
3X	Safor mandarin	*C. hybrid*	IVIA-581
3X	Alborea mandarin	*C. hybrid*	IVIA-592
3X	Coral mandarin	*C. hybrid*	IVIA-593
3X	Tania 46 mandarin	*C. hybrid*	IVIA-594
3X	Matiz mandarin	*C. hybrid*	IVIA-595
4X	Chandler pummelo	*C. maxima*	–
4X	Nadorcott mandarin	*C. reticulata*	–
4X	Moncada mandarin	*C. hybrid*	–
4X	Eureka lemon	*C. limon*	IVIA-495
4X	Cleopatra mandarin	*C. reshni*	IVIA-502
4X	Alemow	*C. macrophylla*	IVIA-518
*The name of the species is based on the Tanaka classification (Tanaka, 1954; Tanaka, 1977).			

Between 40 and 50 flowers in pre-anthesis were collected from each genotype from the four cardinal points of the tree during Spring 2018. The anthers were removed from the flowers and placed in Petri dishes (50–80 anthers/plate, 16–25 plates/genotype) inside a desiccator containing silica gel until the anthers opened within 24–48 h. Petri dishes containing fully dehisced anthers were then sealed with parafilm and stored at −20°C until use (Volk, 2011).

### Pollen Nuclei Isolation and Ploidy Level Analysis

Flow cytometry was used to determine the ploidy level of the control leaves and mature pollen grains. As controls, we used leaves of haploid and diploid clementine (*Citrus clementina* Hort. ex Tan.). Leaf samples were chopped using a razor blade in the presence of a nuclei isolation buffer (Galbraith et al., 1983). Nuclei were filtered through a 30 µm nylon filter and stained with DAPI (1.5 µg/ml). After 10 min incubation, stained samples were run on a BD Influx (BD Biosciences, USA) and analyzed with BD FACS software.

To determine the pollen size, pollen from fully dehisced anthers were distributed with a brush onto a microscope slide. Preparations were observed under a Leica DMLS microscope and the diameters of 200 mature pollen grains were measured using the ImageJ2 software (Schindelin et al., 2015). To analyze the ploidy level of mature pollen grains, pollen nuclei were isolated as described by Kron and Husband (2012). The mesh size of the pre-filter and bursting filter was 50 and 20 µm, respectively. Afterwards, 4–5 dehisced anthers were collected in a 1.5 ml tube adding 300 µl of nuclei isolation buffer. The suspension was vortexed to release all pollen grains. The suspension was then filtered using the pre-filter and the bursting filter (CellTrics^®^ filters, Partec^®^). The bursting filter, with the collected pollen grains, was then placed inside a clean tube and the pollen grains were gently rubbed against the filter for 10–15 s, using a plastic rod. Nuclei were rinsed through the filter with nuclei isolation buffer, and the process was repeated twice. After adding DAPI (1.5 µg/ml) the suspension was incubated for 10 min on ice and run on the cytometer as described before.

### FACS-Based Purification of Single Nuclei and Whole Genome Amplification

FACS-based purification of single nuclei and WGA was carried out following the methodology described by Dreissig et al. (2015). From the nuclei suspension, single nuclei were sorted using a BD Influx cell sorter (BD Biosciences, USA) into individual wells of a 384-microwell plate containing 2 μl lysis solution, which was composed of 0.5 μl lysis buffer, 0.5 μl ddH2O and 1 μl sample buffer (Genomiphi V2, GE Healthcare).

To control the nuclear composition of the individual peaks, nuclei from the different fractions were sorted onto microscopic slides and checked under a fluorescence microscope Axioplan2 (ZEISS, Jena, Germany) equipped with an ORCA-ER CCD camera (Hamamatsu, Japan). Pictures were taken using the Simple PCI (Compix Inc., Imaging Systems, USA) software.

WGA was performed using the Illustra GenomiPhi V2 DNA Amplification Kit (GE Healthcare, USA). Nuclei lysis and DNA denaturation were conducted by incubation at 65°C for 3 min in 2 μl lysis solution. The lysis solution was neutralized by adding 0.5 μl neutralization buffer (666 mM Tris–HCl, 250 mM HCl). Afterwards, a master mix composed of 3.5 μl sample buffer, 4.5 μl reaction buffer and 0.5 μl enzyme mix (Genomiphi V2, GE Healthcare) per reaction was added and samples were incubated at 30°C for 8 h followed by inactivation of the enzyme at 65°C for 10 min. Subsequently, each sample was diluted with 500 μl ddH2O. The DNA concentration of the WGA products was measured by fluorometric quantitation (Qubit, Life Technologies). Additionally, several single pollen nuclei of the genotypes subjected to WGA were mixed in the same well to be used as a positive control against amplification errors (Dreissig et al., 2015).

### Genotyping With Molecular Markers

WGA pollen DNA and genomic leaf DNA were genotyped with SSR and SNP molecular markers displaying heterozygosity for the analyzed genotypes (**Table 2**). These markers are distributed across all linkage groups (LGs) of the clementine genetic map (Ollitrault et al., 2012). Genomic DNA from control leaves was isolated using a Plant DNeasy kit from Qiagen Inc. (Valencia, CA, USA) following the manufacturer’s protocol.

**Table 2:** Information about the molecular markers used in citrus pollen grain and leaf nuclei genotyping, including GenBank accession numbers, genetic distances, noted alleles and bibliographic references.

Genotype	Locus	Gene bank/phytozome accesion	Marker type	Linkage group	Genetic map locus position (cM)	Distance to the centromere (cM)	Noted alleles	Reference
CSO tangor	CIBE6147	ET085226	SSR	1	2.69	57.97	204–212	Ollitrault et al. (2010)
	2P21022555	Ciclev10018135 m.g	SNP	2	57.00	0.10	A:T	Curk et al. (2015)
	MEST470	DY290454	SSR	3	88.76	1.83	254–258	In preparation
	CF-ACA01	CN181701.1	SSR	4	24.41	8.30	335–338	In preparation
	MEST15	FC912829	SSR	5	16.21	6.91	174–192	García-Lor et al. (2012)
	CiC4356-06	ET111465	SNP	6	6.21	0.20	C:T	Ollitrault et al. (2012)
	mCrCIR03B07	FR677573	SSR	7	83.39	13.04	263–265	Cuenca et al. (2011)
	LCY2-M-379	FJ516403	SNP	8	58.10	3.90	A:G	Ollitrault et al. (2012)
Carrizo citrange	SOS1-M50	JX630068	SNP	1	78.51	17.85	A:G	Garcia-Lor et al. (2013a)
	PSY-M30	JX630080	SNP	6	69.72	63.52	C:G	Garcia-Lor et al. (2013a)
	FLS-P129	JX630083	SNP	7	45.99	50.44	C:T	Garcia-Lor et al. (2013a)
Eureka Frost lemon	CiC2110-02	ET099643	SNP	1	29.61	31.05	A:C	Ollitrault et al. (2012)
	CiC3712-01	ET079481	SNP	2	93.92	37.05	A:C	Ollitrault et al. (2012)
	CiC1459-02	ET073328	SNP	3	118.06	27.47	A:C	Ollitrault et al. (2012)
Moncada mandarin	CIC2810-01	ET103230	SNP	1	63.40	2.7	A:C	Ollitrault et al. (2012)
	INVA-P855	JX630071	SNP	3	30.21	60.37	C:T	Garcia-Lor et al. (2013a)
	LAPXCF238	EU719653	SNP	6	19.16	12.96	G:C	Ollitrault et al. (2012)
Mexican lime	CIC6213-07	ET085253	SNP	4	84.57	61.45	G:A	Ollitrault et al. (2012)
	CIC4356-06	ET107540	SNP	6	6.15	0.05	C:T	Ollitrault et al. (2012)
	CHI-M598	JX630075	SNP	4	11.03	5.13	C:G	Garcia-Lor et al. (2013a)

PCR amplification using SSR markers was performed using a Thermocycler rep gradient S (Eppendorf^®^) in 15 μl containing 0.5 μl 1 U/μl of Taq DNA polymerase (Fermentas^®^), 3 μl citrus DNA, 1.5 μl of 2 mM welled (Sigma^®^) dye-labeled forward primer, 1.5 μl of 2 mM non-dye-labeled reverse primer, 0.2 mM of each dNTP, 1.5 μl 10X PCR buffer, and 0.45 μl 50 mM MgCl_2_. The PCR protocol was as follows: denaturation at 94°C for 5 min followed by 40 cycles of 30 s at 94°C, 30 s at 50 or 55°C, and 30 s at 72°C; and a final elongation step of 8 min at 72°C. Capillary electrophoresis was carried out using a Genetic Analysis System 8000 (Beckman Coulter Inc.). The PCR products were initially denatured at 90°C for 2 min, injected at 2 kV for 30 s, and separated at 6 kV for 35 min. Alleles were sized based on a DNA size standard (400 bp). GenomeLab™ v.10.0 (Beckman Coulter Inc.) genetic analysis software was used for data collection.

SNP markers were genotyped using KASPar™ technology by LGC Genomics (Hoddesdon, UK). The KASPar™ Genotyping System is a competitive, allele-specific dual Förster resonance energy transfer (FRET)-based assay for SNP genotyping. Primers were directly designed by LGC Genomics based on the SNP locus flanking sequence. A detailed explanation of the specific conditions and reagents using the KASPar technique can be found in Cuppen (2007).

In order to test if WGA of diploid nuclei is an adequate approach to identify unreduced gametes, 10 individualized nuclei from leaves of “Carrizo” citrange, “Eureka” lemon, “Mexican” lime, and “Moncada” mandarin were isolated and amplified. As positive controls, DNA from leaves and 20 leaf nuclei were isolated from each genotype in the same well. WGA products were evaluated using three different heterozygous SNPs markers for each genotype located in different LGs.

## Results

### Ploidy Level of Mature Pollen Grains of Diploid, Triploid, and Tetraploid Citrus Genotypes

**Figure 1:**
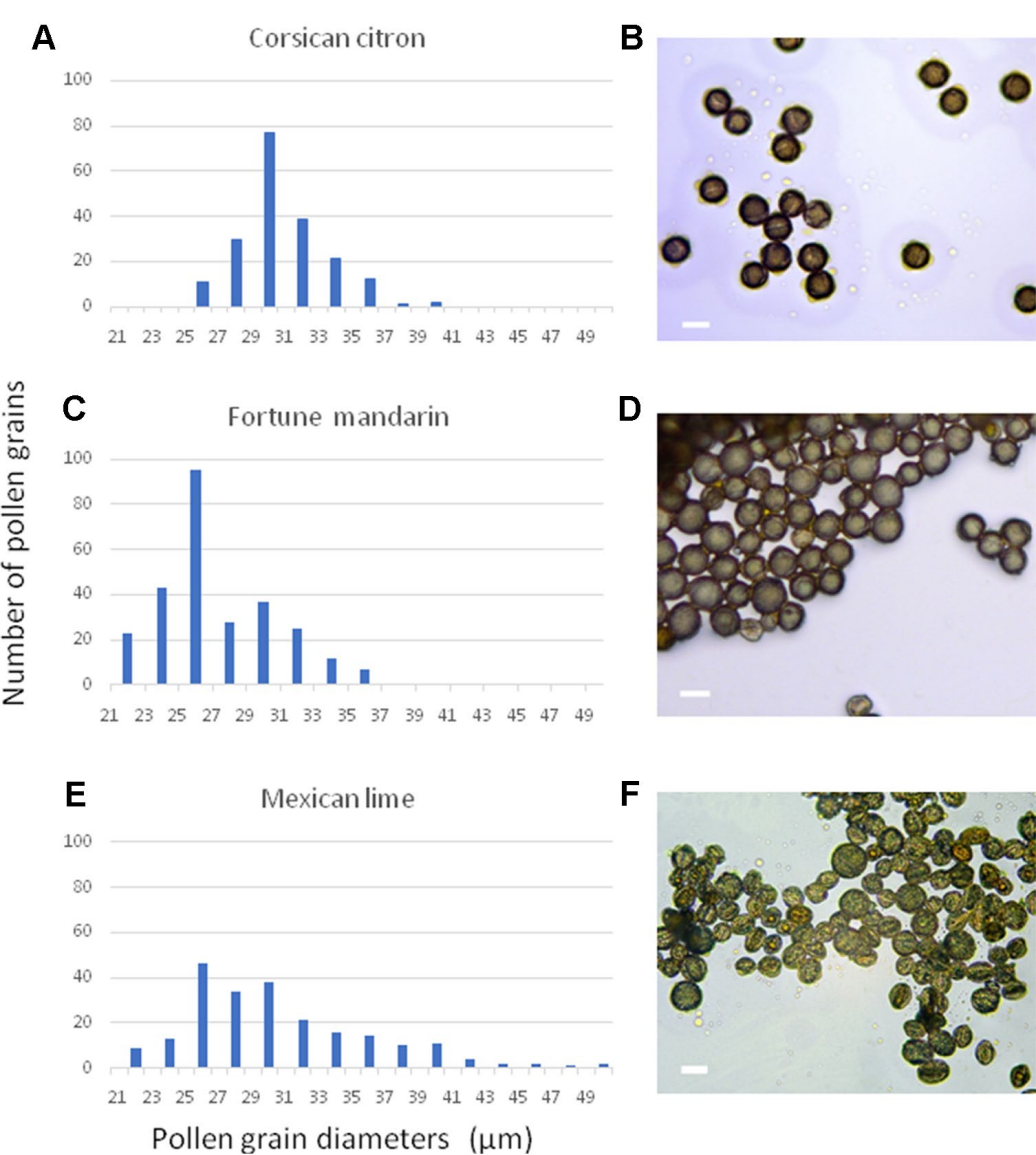
Distribution of representative frequencies and images showing the heterogeneity of pollen diameters in three citrus genotypes: **(A**, **B)** “Corsican” citron, **(C**, **D)** “Fortune”mandarin, **(E**, **F)** “Mexican” lime. Scale bars, 30µm.

To isolate the nuclei of mature citrus pollen grains for flow cytometric measurements of the DNA content a practical and efficient protocol is needed. For this purpose, we tested different methodologies reported in the literature, including mechanical crushing (Galbraith et al., 1983) and sonication (Dewitte et al., 2009). However, these approaches resulted in high levels of background and no conspicuous and reliable peaks were detected in the histograms (data no shown). Only the “bursting” methodology proposed by Kron and Husband (2012) resulted in clear DNA peaks. To determine the necessary diameters of the pre- and bursting filters required for the pollen grain and nuclei isolation, respectively, we measured the diameters of pollen grains of 10 different citrus genotypes representing ancestral and secondary species. **Figure 1** shows selected examples of pollen grain size measurements of the cultivars “Corsican” citron, “Fortune” mandarin and “Mexican” lime. The populations of pollen grains showed a unimodal distribution, with slight differences in sizes between genotypes. The diameters of “Corsican” citron pollen grains ranged from 26 to 40 µm, between 22 and 36 µm for “Fortune” mandarin and “Mexican” lime showed a size distribution between 22 and 50 µm (**Figure 1**). The minimum diameter value observed was 22 μm and the maximum value was 50 μm; therefore, we decided to work with the 50 μm pre-filter and the 20 μm bursting filter. Then, following this methodology, we obtained histograms with relatively low background and distinct peaks.

**Figure 2:**
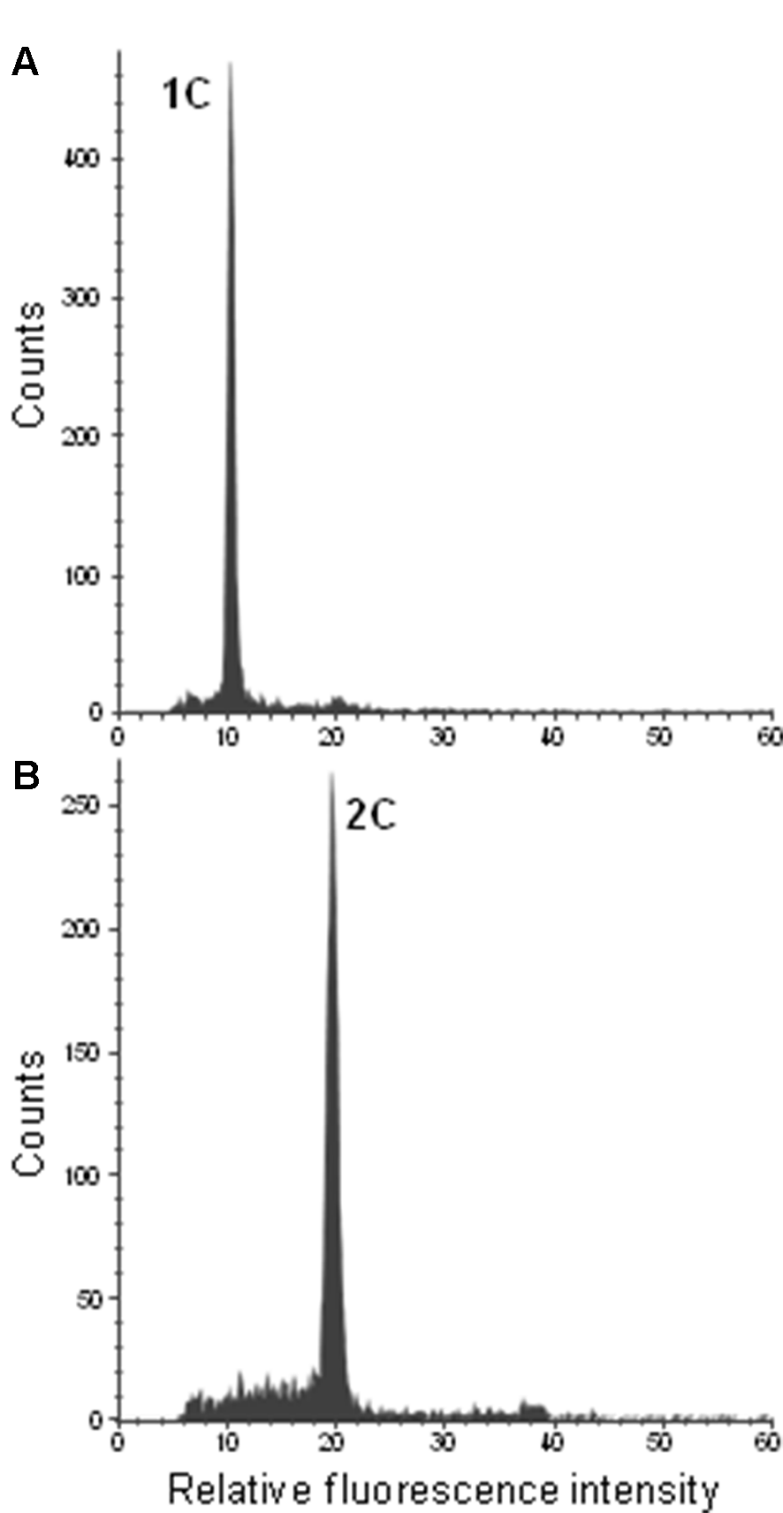
Flow cytometric histograms of DAPI-stained leaf nuclei of the haploid control plant IVIA-638 **(A)** and the diploid control plant IVIA-039 **(B)** of clementine.

**Figure 3:**
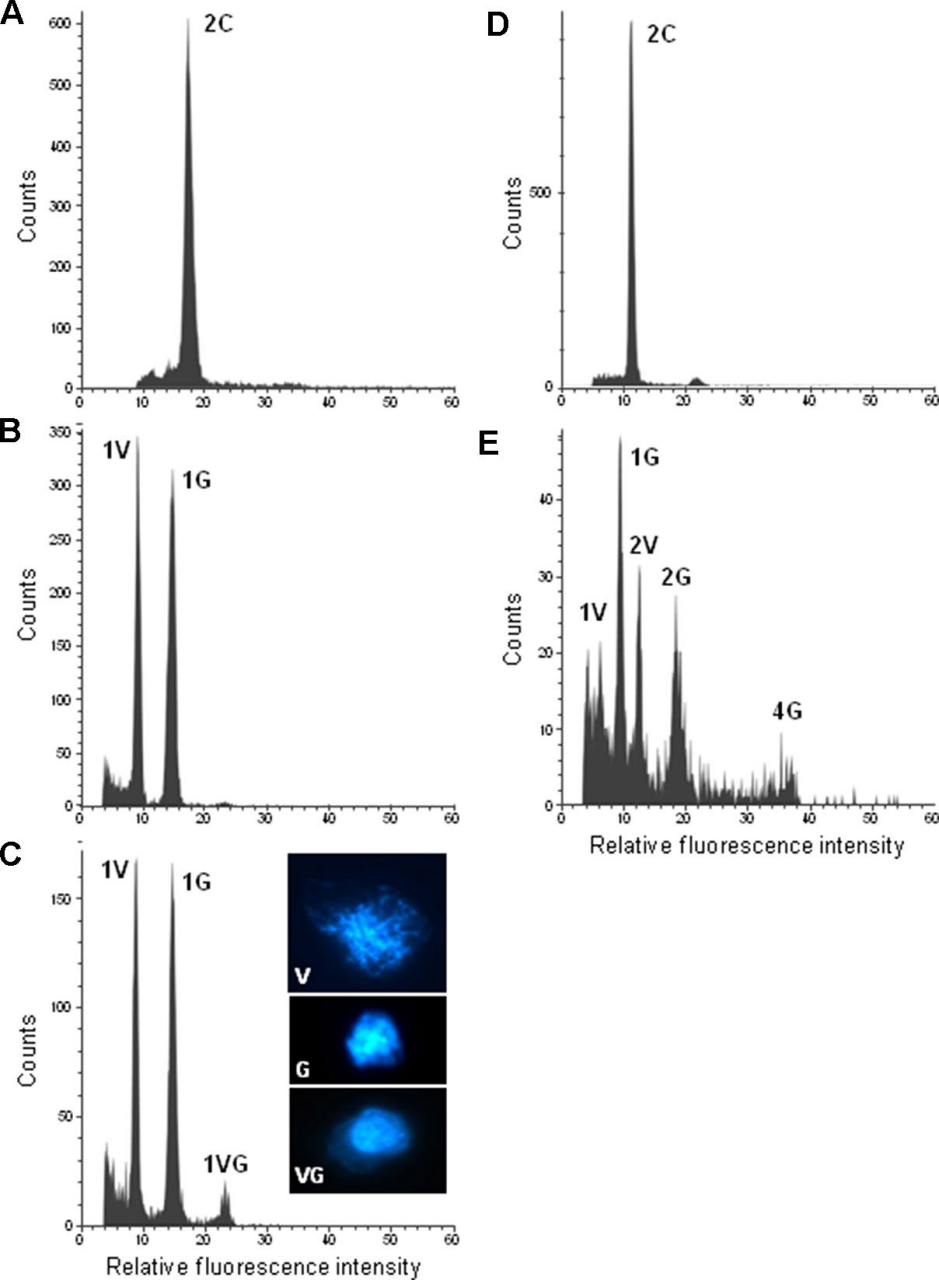
Flow cytometric measurements of DAPI-stained leaf and pollen nuclei of diploid citrus genotypes: **(A)** “CSO” tangor leaf. **(B)** “Moncada” mandarin pollen. **(C)** “CSO” tangor pollen. **(D)** “Mexican” lime leaf. **(E)** “Mexican” lime pollen. Inserts in c show examples of flow sorted pollen nuclei of the corresponding histogram peaks of “CSO” tangor. V, G and VG represent vegetative, generative and vegetative plus generative pollen nuclei, respectively. Please note that the histograms of “CSO” tangor and “Mexican” lime were recorded on different days with different cytometer settings resulting in a variation in the peak positions when both genotypes are compared with each other.

To confirm ploidy differences between the selected *Citrus* genotypes, we performed comparative measurements of nuclei isolated from leaf tissue of two different clementine genotypes. In both cases, only one dominant peak was observed with the diploid genotype (IVIA-039) showing the peak at a fluorescence value twice as big as that of the haploid genotype (IVIA-638) (**Figure 2**). Subsequently, we analyzed the ploidy level of mature pollen grains of 45 diploid, 7 triploid, and 6 tetraploid genotypes (**Table 1**). Forty-three of the 45 diploids showed histograms with two peaks (**Figure 3B**), corresponding to the 1C peak of the vegetative nuclei (V) and the 2C peak of the generative nuclei as it is typical for species with binucleate pollen (Van Tuyl et al., 1989; Bino et al., 1990; Kron and Husband, 2012). However, exceptions were observed for “CSO” tangor and “Mexican” lime. Pollen from the “CSO” tangor produced a histogram with an additional third peak (**Figure 3A–C**). The fluorescence values of the first two nuclei populations coincide with the values obtained for the other diploid genotype analyzed (1V, 1G), whereas the third peak (1VG) displayed a higher fluorescence intensity than the other two peaks (**Figures 3B, C**). To identify the origin of this third peak, we sorted nuclei corresponding to the three individual peaks separately and evaluated them microscopically. Nuclei from the populations 1V and 1G revealed different chromatin structures. While the population with the lower fluorescence intensity corresponding to the vegetative nuclei (1V) revealed a relaxed chromatin condensation, the generative nuclei (1G) showed a condensed chromatin structure (**Figure 3C**). Interestingly, the third peak (1VG) reflects two nuclei attached to each other, one with a relaxed structure and one with a compact structure (**Figure 3C**).

On the other hand, pollen nuclei from “Mexican” lime showed five populations (**Figure 3D, E**). Beside 1V and 1G populations, which were also present in the other diploid genotypes analyzed; we found two additional populations with twice the fluorescence value of 1V and 1G nuclei populations which were named 2V and 2G, respectively (2V/1V = 1.98; 2G/1G = 1.98); and a fifth small peak with twice the fluorescence of the 2G peak, named 4G, with a correlation in the DNA content of the 4G and 2G populations (4G/2G = 1.94). The fluorescence ratios between 2V/1V and 2G/1G indicate that 2V and 2G peaks potentially contain unreduced gametes, whereas the 4G peak might correspond to a population of tetraploid nuclei that could be originated as a consequence of doubled-unreduced gametes. However, due to a low number of viable pollen grains in “Mexican” lime (Pons et al., 2011; Rouiss et al., 2018), the number of nuclei of the corresponding ploidy levels is relatively low and the resulting peaks not very pronounced.

**Figure 4:**
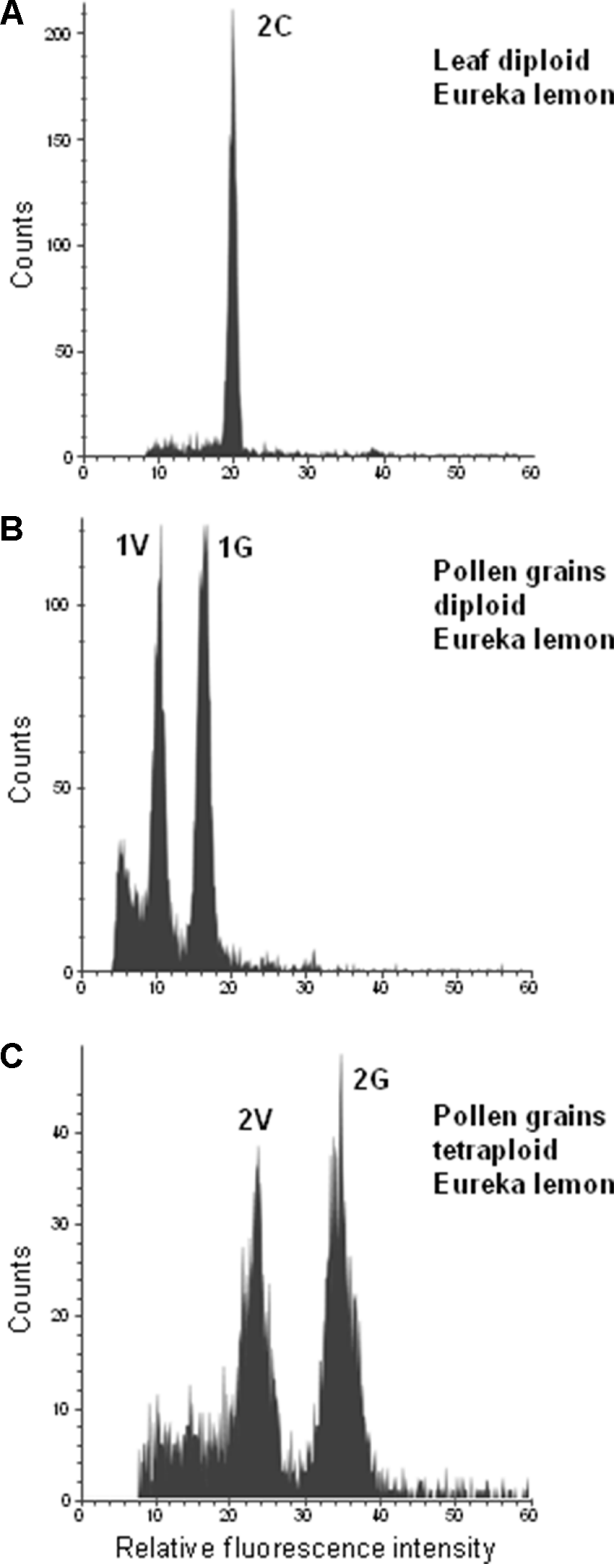
Flow cytometric measurements of leaf and pollen nuclei of “Eureka” lemon. **(A)** Diploid “Eureka” lemon leaf, **(B)** Pollen of Diploid “Eureka” lemon. **(C)** Pollen of tetraploid “Eureka” lemon.

The tetraploid genotypes showed two populations of nuclei with fluorescence values twice as high as that of the populations of the 1V and 1G nuclei of diploid genotypes (**Figure 4**). Nuclei of pollen grains of triploid genotypes could not be isolated (data not shown).

### FACS, WGA, and Genotyping of Single Pollen Nuclei

In order to determine the genotype underlying the three different populations of pollen nuclei found in “CSO” tangor, FACS-based isolation of single nuclei coupled with whole-genome amplification was performed. A total of 72 nuclei were isolated, 24 nuclei from each of the three fluorescence peaks (1V, 1G and 1VG, **Figure 3C**), along with 20 nuclei sorted into the same well as positive control and leaf DNA. To confirm the successful sorting of the nuclei in single microwells and to identify their genetic origin, WGA products were analyzed with five SSR and three SNP markers heterozygous for “CSO” tangor (**Table 3**) located in eight different linkage groups (LGs) of th e clementine genetic map (Ollitrault et al., 2012). From the 72 nuclei, 63 displayed at least 50% of positive PCR reactions (87.5%). Out of 576 PCR reactions for all marker combinations, 487 were positive (84.5%).

**Table 3:** Results by linkage group (LG) and molecular marker of the diploid genotype “CSO” for populations of vegetative nuclei 1V, generative nuclei 1G and attached nuclei 1VG.

Samples		Molecular markers by Linkage group in brackets															
		CIBE6147 (1)		2P21022555 (2)		MEST470 (3)		CF-ACA01 (4)		MEST15 (5)		CiC4356-06 (6)		mCrCIR03B07 (7)		LCY2-M379 (8)	
Leaf control		204–212		AG		254–258		336–339		174–192		TC		263–265		GA	
PPC^1^		204–212		AG		254–258		336–339		174–192		TC		263–265		GA	
V-01		–		G		254		336		192		C		263		A	
V-02		–		A		–		–		174		C		265		A	
V-03		212		G		–		336		174		T		265		G	
V-04		204		G		254		–		192		C		263		–	
V-05		–		–		–		–		–		–		–		–	
V-06		–		G		254		336		192		T		263		G	
V-07		–		–		–		–		–		–		–		–	
V-08		212		G		–		339		174		T		265		A	
V-09		–		–		–		–		–		C		–		–	
V-10		212		A		–		336		192		C		263		G	
V-11		212		G		258		336		174		C		263		A	
V-12		204		A		254		336		192		C		263		A	
V-13		204		–		254		339		192		–		–		–	
V-14		204		A		254		336		192		C		263		–	
V-15		204		G		254		336		174		C		263		G	
V-16		204		G		258		336		192		C		263		G	
V-17		212		G		258		339		192		C		265		G	
V-18		–		–		–		–		–		–		–		–	
V-19		212		G		258		336		174		T		265		A	
V-20		204		G		254		339		192		T		263		G	
V-21		–		–		–		–		–		–		–		–	
V-22		–		–		–		–		–		C		–		–	
V-23		212		G		258		339		192		C		265		G	
V-24		212		–		254		336		174		T		265		A	
G-01		204		A		258		339		174		C		263		A	
G-02		204		A		254		336		192		T		265		G	
G-03		–		A		–		–		–		–		–		–	
G-04		204		A		254		336		192		T		263		G	
G-05		212		G		258		339		192		C		263		G	
G-06		212		G		258		339		174		C		265		G	
G-07		204		G		254		339		192		C		263		G	
G-08		204		G		258		339		174		T		263		G	
G-09		–		–		–		–		–		–		–		–	
G-10		–		G		–		–		174		C		265		A	
G-11		204		G		254		336		174		C		263		A	
G-12		204		A		254		339		192		C		263		G	
G-13		204		G		258		339		192		C		263		A	
G-14		204		G		254		–		192		T		263		A	
G-15		212		A		254		339		192		T		265		G	
G-16		204		A		254		336		192		C		263		G	
G-17		212		G		258		339		192		T		265		G	
G-18		212		G		258		339		174		T		265		A	
G-19		212		A		254		339		192		T		263		A	
G-20		204		–		258		336		192		T		265		A	
G-21		204		G		258		336		192		C		265		G	
G-22		212		G		254		339		174		T		265		A	
G-23		212		G		254		339		174		T		265		G	
G-24		204		G		258		339		174		T		265		–	
VG-01		212		A		258		336		174		T		263		A	
VG-02		204		A		258		336		192		C		265		A	
VG-03		212		A		258		336		192		T		263		G	
VG-04		212		G		254		336		174		T		263		A	
VG-05		212		G		258		336		192		T		263		A	
VG-06		204		G		254		336		174		T		263		A	
VG-07		212		–		–		339		–		–		265		–	
VG-08		204		G		254		336		174		C		265		A	
VG-09		212		A		254		336		174		C		265		A	
VG-10		212		G		254		336		192		C		265		G	
VG-11		212		A		254		336		192		C		265		A	
VG-12		212		G		254		336		174		C		265		G	
VG-13		212		G		254		336		174		T		265		A	
VG-14		212		G		254		336		174		C		263		G	
VG-15		204		A		258		336		174		C		265		G	
VG-16		204		G		258		336		192		C		265		A	
VG-17		212		G		258		336		–		T		–		G	
VG-18		212		G		254		336		192		T		263		G	
VG-19		212		G		258		336		192		C		265		A	
VG-20		204		G		254		336		174		C		263		A	
VG-21		212		G		258		339		192		C		263		A	
VG-22		212		G		254		336		192		T		263		G	
VG-23		212		G		258		339		192		T		263		G	
VG-24		212		A		254		336		192		C		265		A	

^1^PPC, Pollen positive control.

V samples correspond to nuclei isolated from the 1V fluorescence peak, G from the 1G fluorescence peak and VG from the 1VG fluorescence peak.

The marker call rates were 68.2% for the 1V nuclei population, 91.7% for the 1G nuclei population and 95.8% for the 1VG population (**Table 3**). These results could be related with the increasing number of copies in each nuclei population. The marker analysis displayed heterozygosity for the leaf and pollen positive controls, as expected (**Table 3**). In contrast, only one allele was observed with all SSR and SNP markers for the 1V, 1G, and 1VG nuclei populations. Therefore, we confirmed that the three nuclei populations recovered from pollen grains of the diploid “CSO” tangor are based on a single allele of the same origin each. In addition, the fact that the nuclei population with the highest fluorescence peak (1VG) is homozygous reflects that this population is composed of two attached nuclei (1V+1G) derived from the same pollen grain.

Taken together the results obtained with molecular markers for “CSO” tangor and the histogram obtained after ploidy level analysis of mature pollen grains of “Mexican” lime, we can consider that the peaks 1V and 1G in “Mexican” lime correspond to vegetative and generative nuclei, whereas 2V and 2G peaks correspond to unreduced gametes.

**Figure 5:**
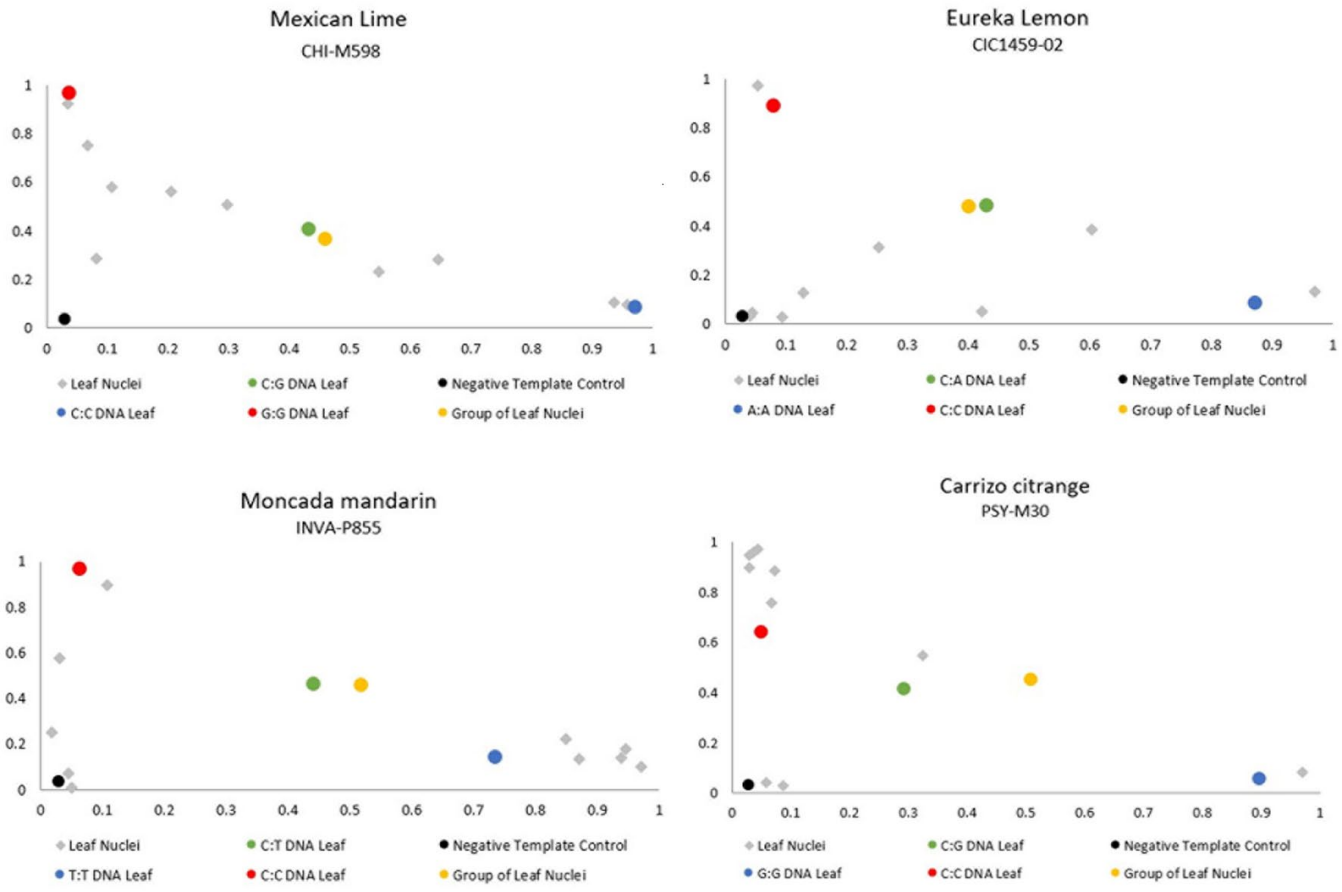
Representative dispersion diagram of PCR products of the leaf nuclei amplified with the WGA kit of the four diploid genotypes analyzed.

We tested the genotyping of WGA-diploid nuclei as an approach to identify unreduced gametes by isolating and amplifying individualized nuclei from leaves of several genotypes, along with DNA from leaves and 20 leaf nuclei mixed from each genotype. Out of the 40 amplified nuclei, 24 produced positive PCR amplifications for at least 50% of the analyzed markers (62.5%). The amplification ratios were 70% for “Carrizo” citrange, 90% for “Mexican” lime and 60% for “Eureka” lemon and “Moncada” mandarin. For these leaf nuclei, the ratios of the allele signals for all marker-genotype combinations are scattered in the plot, without forming a clear heterozygous group, as expected, and even showing homozygous amplifications. For example, DNA from leaves and leaf nuclei positive control of “Mexican” lime genotyped with the CHI-M598 SNP marker, resulted in heterozygous call rates (CG alleles), as expected. Furthermore, we used DNA from homozygous leaf controls, producing homozygosity for each allele, CC and GG signals. However, leaf nuclei amplifications displayed no defined heterozygous clusters and even some amplifications are grouped together with the homozygous controls (**Figure 5**). These results show that the WGA kit in at least some cases only amplifies a single allele of the two present alleles or the allele amplification is not balanced (**Figure 5**). With these results, the WGA of diploid nuclei of unreduced gametes of “Mexican” cannot be used for genotyping and to identify the mechanism underlying the formation of 2n pollen gametes.

## Discussion

### Flow Cytometry Is a Simple and Efficient Method to Determine the Ploidy Level of Citrus Pollen Grains

The applications of flow cytometry has increased considerably since its inception (Doležel et al., 2007; Orbović et al., 2008; Adan et al., 2017; Bourge et al., 2018; Hoang et al., 2019). This method has become a reliable and fast tool in plant biology to determine the nuclear DNA content (Kron and Husband, 2012; Hoang et al., 2019) as well as ploidy levels (Eeckhaut et al., 2005; Ochatt, 2008; Bourge et al., 2018). In citrus, flow cytometry has been mainly used to determine the genome size and the ploidy level of somatic tissue in many breeding programs (Ollitrault and Michaux-Ferrière, 1992; Seker et al., 2003; Guo et al., 2004; Orbović et al., 2008; Aleza et al., 2009a; Dutt et al., 2010; Cuenca et al., 2011; Kamiri et al., 2011; Aleza et al., 2012a; Aleza et al., 2012b; Navarro et al., 2015; Rouiss et al., 2017a; Rouiss et al., 2017b; Kamiri et al., 2018).

In recent years, flow cytometry has also been used to study the DNA content of pollen nuclei in a large number of plant genera, which opens up new opportunities to study the meiotic processes, such as sexual polyploidization (Dewitte et al., 2009; Kron and Husband, 2015; Sora et al., 2016; Kreiner et al., 2017;), and demonstrating that many angiosperms are capable of producing 2n gametes (Van Tuyl et al., 1989; Pan et al., 2004; Kron and Husband, 2012; Chung et al., 2013). In this paper, we apply for the first time flow cytometry to study pollen grain populations in several species of the genus *Citrus*. Our study confirms that flow cytometry as a simple and cost-effective methodology to determine the ploidy level of citrus pollen grains. Quality histograms were obtained with DAPI-stained pollen nuclei, as reported for other species (Van Tuyl et al., 1989; Pan et al., 2004; Doležel et al., 2007; Dewitte et al., 2009; Kron and Husband, 2012).

The DNA content of haploid leaf nuclei is expected to be similar to that of the vegetative pollen nuclei, as their ploidy level is x (in the case of *Citrus* x = 9), while the DNA content in diploid nuclei from leaf is expected to be similar to that of the generative pollen nuclei, as their DNA content is 2C. However, we have observed slightly lower fluorescence for the generative pollen nuclei in comparison with leaf nuclei. These differences have also been reported for other species (Van Tuyl et al., 1989; Bino et al., 1990; Dewitte et al., 2009; Kron and Husband, 2012). The reason behind could be structural differences between the generative and leaf nuclei (Bennett and Leitch, 2005) and fluorescence inhibition that operates differently on leaf and the pollen nuclei (Price et al., 2000).

We confirmed by microscopy and flow cytometry that mature pollen grains of citrus are binucleated. Histograms of the analyzed pollen grains from diploid donors revealed the clear presence of two populations with different fluorescence intensities, which agrees with what was found for pollen grains of other diploid angiosperm species (Van Tuyl et al., 1989; Bino et al., 1990; Pan et al., 2004; Dewitte et al., 2009; Kron and Husband, 2012). The first population contained vegetative nuclei, with half of the DNA content of leaf nuclei whereas the second population composed of generative nuclei has almost the same DNA content as the leaf tissue. This indicates that the generative nucleus is in the post replication stage of the cell division (G2) (Van Tuyl et al., 1989; Bino et al., 1990; Dewitte et al., 2009; Kron and Husband, 2012; Kron and Husband, 2015). Afterwards, the generative nuclei performs a further mitotic division to produce twin sperm cells responsible for the double fecundation (Berger and Twell, 2011). Citrus pollen contained nuclei having structural differences. The vegetative nucleus is large and less condensed, while the generative nucleus is more condensed. These differences have also been observed in other species (Dewitte et al., 2009; Alonso Pen~a, 2011; Kron and Husband, 2015). Only two exceptions were found in the analyzed histograms of the diploid genotypes “CSO” tangor and “Mexican” lime, in which beside 1V and 1G peaks additional peaks were found.

In “CSO” tangor, we observed an additional peak named 1VG, (**Figure 3C**). The fluorescence value of this peak suggests that the vegetative and generative nuclei of the same cell remained attached to each other after the opening of the pollen using the bursting-isolation method. In addition, we confirmed with SSR and SNPs markers that the each of the three nuclei populations has an identical genetic origin. On the other hand, Rouiss et al. (2017a) identified the presence of unreduced pollen from the diploid genotype “CSO” by analyzing tetraploid progenies obtained with a tetraploid female parent. However, we did not find any peak corresponding to unreduced pollen. In this context, in addition to genetic factors, the generation of unreduced gametes is also influenced by environmental factors (Souza et al., 2004; d’Erfurth et al., 2009; Dewitte et al., 2009; Aleza et al., 2010; Mason et al., 2011; De Storme et al., 2012; De Storme and Geelen, 2013; Sora et al., 2016; Wang et al., 2017), which might not be favoring their production during the season covered by the present study. In fact, tetraploid progenies from “CSO” were only obtained during the season in which Rouiss et al. (2017a) performed their study (H. Rouiss, personal communication). Nevertheless, our methodology could be applied to further studies focused on how temperature stress or environmental conditions could influence the formation of 2n gametes in citrus.

Unreduced pollen grains were observed in the diploid “Mexican” lime. To our knowledge, this is the first report on the observation of this type of pollen population by flow cytometry in *Citrus*. The “Mexican” lime pollen showed 2V and 2G nuclei populations, with similar fluorescence intensit as the vegetative and generative pollen nuclei of tetraploid genotypes. The presence of pollen nuclei with a duplicated DNA content (2G) in comparison to leaf nuclei (2C) was observed for other plant genera before (Van Tuyl et al., 1989; Bino et al., 1990; Dewitte et al., 2009). In addition, a 4G nuclei population was observed with a double DNA content of the 2G population which could be due to the formation of doubled-unreduced gametes. This phenomenon is unusual although the formation of tetraploid pollen due to the lack of post-meiotic cytokinesis in all microspore mother cells has been described in alfalfa (McCoy and Smith, 1983; Pfeiffer and Bingham, 1983; Tavoletti et al., 2000). Doubled-unreduced gametes have been observed in interspecific strawberry hybrids (Bringhurst and Gill, 1970). We also observed large pollen grains in this genotype (**Figure 1F**). The presence of enlarged pollen grains is generally associated with the formation of unreduced gametes (Jones et al., 2007; Honsho et al., 2016). The “Mexican” lime is a interspecific hybrid between *C. micrantha* × *C. medica* (Nicolosi et al., 2000; Garcia-Lor et al., 2013b; Curk et al., 2016). It is known that interspecific hybrids of other plant genera show a tendency towards the formation of unreduced gametes (Van Tuyl et al., 1989; Ramanna et al., 2003; Mason et al., 2011; Chung et al., 2013; Martin et al., 2019). In addition, Curk et al. (2016) proposed that *C. latifolia* (“Tahiti” lime like accessions) resulted from the fertilization of a haploid lemon ovule by a diploid gamete of a diploid “Mexican” like lime. These observations support the hyphothesis that “Mexican” lime produce unreduced pollen gametes.

In contrast to all diploid and tetraploid genotypes, the results obtained from the analysis of the triploid genotypes showed no clear peaks in the histograms. In general, citrus triploid hybrids do not produce seeds or induce seeds in other genotypes by cross-pollination since they have very low male and female fertility (Aleza et al., 2010; Aleza et al., 2012a; Phillips et al., 2016). Odd-numbered polyploids oftenhave pollen of high rates of infertility or aneuploidy, as reported for other species (e.g. *Aeschynanthus* spp., *Alstroemeria* spp., *Crocus* spp., *Lilium* spp.; Rounsaville et al., 2011; Kron and Husband, 2012; Phillips et al., 2016). The absence of clear peaks in the histograms has been demonstrated to be due to pollen infertility.

Our flow cytometry analysis of pollen from tetraploid genotypes revealed the presence of two populations, i.e., vegetative and generative pollen nuclei, both showing twice the fluorescence than their counterpart nuclei in the diploid genotypes. Although cases of unreduced pollen have been reported in diploid citrus genotypes (Honsho et al., 2016; Rouiss et al., 2017a), little is known regarding tetraploid genotypes. In studies of progenies from controlled 2X × 4X hybridizations, where the female parent is self-incompatible, double reduced haploid pollen gametes were observed, resulting in diploid plants (Aleza et al., 2012a). Kamiri et al. (2009) also reported the production of haploid gametes from citrus allotetraploid somatic hybrids. Koutecký et al. (2011) observed one pentaploid hybrid between diploid *Centaurea pseudofirgia* and tetraploid *C. jacea*, arising from a reduced diploid ovule and a tetraploid unreduced pollen grain. These studies showed that tetraploid genotypes can produce pollen grains with different ploidy levels than the expected, and these variants are difficult to identify by flow cytometry.

### FACS Coupled With WGA Is an Effective Tool for the Genotyping of Individualized Pollen Nuclei

We demonstrate that the FACS strategy can be applied to isolate individual nuclei of citrus, allowing their classification and further amplification and genotyping. The use of WGA for individualized nuclei combined with genotyping provides the opportunity to use a large number of molecular markers (Dreissig et al., 2015; Gawad et al., 2016; Dreissig et al., 2017).

The results obtained with the molecular markers allowed us to conclude that the 1V, 1G, and 1VG nuclei populations observed in the “CSO” genotype correspond to reduced and not to unreduced pollen. These findings are of importance for the interpretation of the histograms. We can affirm that, in citrus, the presence of a second peak in pollen from diploid and tetraploid genotypes does not imply their correspondence with 2n pollen, and are in agreement with binuclated citrus pollen grains. To our knowledge, this is the first report in citrus demonstrating that vegetative and generative nuclei have the same genetic configuration based on molecular markers as expected. In this context, vegetative and generative nuclei populations have been identified in different woody and herbaceous species (Van Tuyl et al., 1989; Bino et al., 1990; Dewitte et al., 2009; Kron and Husband, 2012; Kreiner et al., 2017), but their genetic constitution has not been demonstrated.

The use of the WGA method and further genotyping with SSR and SNPs markers shows to be reliable for the genotyping of haploid nuclei isolated from mature pollen grains, offering new approaches to increase our knowledge about citrus genetics. However, the amplifications of diploid leaf nuclei did not show the expected results. WGA of those nuclei and their genotyping with heterozygous markers resulted in unbalanced amplifications, losing the 1:1 expected ratio and biased towards one of the two alleles. This allelic imbalance has been previously observed in single-cell genomic sequencing studies also, which is attributed to the bias in the whole genome amplification that occurs in the initial reaction, which is exaggerated by further reactions (de Bourcy et al., 2014; Gawad et al., 2016; Yuan et al., 2018).

## Conclusions

In this paper, we have demonstrated that flow cytometry is an efficient tool for the analysis of pollen grain nuclei populations in several citrus species. In addition, we report for the first time, the generation of unreduced pollen from the interspecific diploid “Mexican” lime. This methodology opens up new applications in citrus research studies, such as the determination if unreduced pollen frequency in breeding parents and the analysis of environmental conditions (e.g. temperature) on the frequency of the unreduced pollen. Sorted nuclei can be used for subsequent WGA for further genetic studies. The whole genome amplified samples can be used to perform studies related to allele segregation, genetic mapping and meiotic crossovers, without the interference that can be encountered when analyzing the derived progenies. However, WGA from diploid leaf nuclei was generally biased towards one allele, thus hampering the genetic analysis of unreduced pollen and of normal diploid pollen arising from tetraploid parents.

Genotyping of the whole genome amplified DNA derived from single pollen nuclei with SSR and SNP molecular markers resulted in a call rate comparable with those previously reported for several species. The obtained results allowed the genetic analysis of single pollen nuclei arising from diploid parents. Altogether, the methodology presented here represents a very useful tool for facilitating research focused on assessing the genetic origin, evolution, and reproductive biology of citrus populations.

## Data Availability

All relevant data is contained within the manuscript.

## Author Contributions

MG and PA conceived the study and were in charge of the direction and planning. JF isolated pollen nuclei and conducted flow-sorting. SD performed whole genome amplification from single pollen nuclei. MG, JC, and PA genotyped pollen nuclei and analyzed the data. MG, JC, and PA took the lead writing the manuscript with input and review of SD, JF, and AH. All authors read and approved the final version of this manuscript.

## Funding

This work was supported by the project RTA2015-00069-00-00 from the Ministry of “Economía y Competividad” and “Instituto Nacional de Investigación y Tecnología Agraria y Agroalimentaria” and the grant “Programa de Perfeccionamiento” resolution 1177/14 from Consejo Directivo of Instituto Nacional de Tecnología Agropecuaria (Argentina).

## Conflict of Interest Statement

The authors declare that the research was conducted in the absence of any commercial or financial relationships that could be construed as a potential conflict of interest.
